# Automated 4D flow MRI pipeline for the quantification of advanced hemodynamic parameters in the left atrium

**DOI:** 10.1038/s41598-025-34972-7

**Published:** 2026-01-16

**Authors:** Xabier Morales, Ayah Elsayed, Debbie Zhao, Filip Loncaric, Ainhoa Aguado, Mireia Masias, Gina Quill, Marc Ramos, Adelina Doltra, Ana García-Alvarez, Marta Sitges, David Marlevi, Alistair Young, Martyn Nash, Bart Bijnens, Oscar Camara

**Affiliations:** 1 BCN MedTech, Department of Engineering, Universitat Pompeu Fabra, Barcelona, Spain; 2 Auckland Bioengineering Institute, University of Auckland, Auckland, New Zealand; 3 Faculty of Health and Environmental Sciences, Auckland University of Technology, Auckland, New Zealand; 4 Department of Engineering Science and Biomedical Engineering, University of Auckland, Auckland, New Zealand; 5 Cardiovascular Institute, Hospital Clínic, Universitat de Barcelona, Barcelona, Spain; 6 Centre for Biomedical Research on CardioVascular Diseases (CIBERCV), Institut d’investigacions biomèdiques August Pi i Sunyer (IDIBAPS), Barcelona, Spain; 7 Institució Catalana de Recerca i Estudis Avançats, (ICREA), Barcelona, Spain; 8 University Hospital Centre Zagreb, Zagreb, Croatia; 9 School of Biomedical Engineering & Imaging Sciences, King’s College London, London, UK; 10 Institute for Medical Engineering and Science, Massachusetts Institute of Technology, Cambridge, MA USA; 11 Department of Molecular Medicine and Surgery, Karolinska Institutet, Stockholm, Sweden; 12 Centro Nacional de Investigaciones Cardiovasculares (CNIC), Madrid, Spain

**Keywords:** 4D flow MRI, Left atrium, Hemodynamics, Deep learning, Computational pipeline, Left ventricular diastolic dysfunction, Computational biology and bioinformatics, Engineering, Mathematics and computing, Medical research

## Abstract

The left atrium (LA) plays a pivotal role in modulating left ventricular filling, yet its hemodynamics remain poorly understood due to the limitations of conventional ultrasound analysis. Four-dimensional flow magnetic resonance imaging (4D Flow MRI) holds promise for enhancing our understanding of atrial hemodynamics, but its analysis is hindered by the inherently low velocities within the chamber and the modest spatial resolution of 4D Flow MRI. Heterogeneity in acquisition protocols and MRI vendors, and the lack of standardized computational frameworks further complicates the creation of large, comparable datasets needed to assess the prognostic value of hemodynamic markers provided by 4D Flow MRI. To address these challenges, we introduce a computational framework tailored to the analysis of 4D Flow MRI in the LA, enabling the qualitative and quantitative analysis of advanced hemodynamic parameters (e.g., kinetic energy, vorticity, and pressure). We applied this framework to a diverse cohort spanning different degrees of left ventricular diastolic dysfunction to investigate the prognostic potential of these metrics. Our framework proved robustness across multicenter data of varying quality, producing high-accuracy automated segmentations. Notably, our findings show that 4D Flow MRI-derived parameters provide superior differentiation between healthy and pathological states than those available to conventional hemodynamic analysis tools.

## Introduction

The left atrium (LA) plays a pivotal role in modulating left ventricular filling. Although the left ventricle (LV) was long held as the sole determinant of cardiac health prognosis, alterations in LA structure, function, and hemodynamics are now recognized as pivotal factors in various cardiovascular disorders, including atrial fibrillation (AF), heart failure, and ischemic and valvular heart disease^1,2^. For instance, AF-related hemodynamic disruption of the LA can lead to thrombus formation, increasing the risk of cerebrovascular thromboembolic events^3^. Additionally, abnormalities in LA hemodynamics often precede the clinical manifestation of LV diastolic dysfunction (LVDD), which remains difficult to characterize non-invasively^4,5^.

Despite its importance, LA hemodynamics has garnered less attention than more prominent structures such as the LV or the aorta^6^. This issue is compounded by the fact that spectral Doppler, the clinical gold standard for flow assessment, is inadequate to characterize the intricate 3D hemodynamics present in the LA^7^, as it can only provide velocity in one dimension along the beam line. In addition, precise measurements of the pulmonary veins (PV) through transthoracic echocardiography (TTE), relevant for the diagnosis of LVDD, become extremely challenging due to their far-field location^8^.

3D time-resolved phase-contrast magnetic resonance imaging (MRI), widely known as 4D Flow MRI, is a promising cardiovascular imaging modality for comprehensive in vivo hemodynamic studies. It acquires time-resolved 3D blood velocity with full volumetric coverage, allowing retrospective flow quantification in a single non-invasive examination^9^. This enables scrutiny of intrinsically three-dimensional structures such as vortices^10^ and concurrent analysis of all four PV, seldom possible with conventional TTE analyses^11^.

However, the use of 4D Flow MRI in the LA is severely hampered by the low blood velocities present and the low spatial resolution necessary to maintain reasonable acquisition times. Moreover, existing commercial and free 4D Flow MRI analysis pipelines primarily focus on aortic or ventricular flow^12^. The heterogeneous nature of 4D flow MRI acquisition, characterized by varying spatiotemporal resolution and noise levels among different MRI vendors, magnetic field strengths, acquisition protocols, and contrast utilization further complicates standardization of analysis pipelines necessary to amass large, multicenter cohorts. Consequently, few studies have utilized 4D Flow MRI to investigate LA hemodynamics^13–19^ and even fewer have thoroughly explored the wide array of novel hemodynamic parameters as potential prognostic biomarkers.

In light of these challenges, this study introduces an automated computational pipeline specifically designed for the analysis of 4D Flow MRI in the LA. This pipeline can handle multicenter data of varying quality, and enables comprehensive visualization of LA hemodynamics and accurate quantification of advanced 4D Flow MRI hemodynamic parameters. Furthermore, this pipeline fully relies on open-source code and software aiming to make this tool accessible to the wider clinical community, encouraging further research and innovation in LA hemodynamics to improve understanding and treatment of related conditions.

**Fig. 1:**
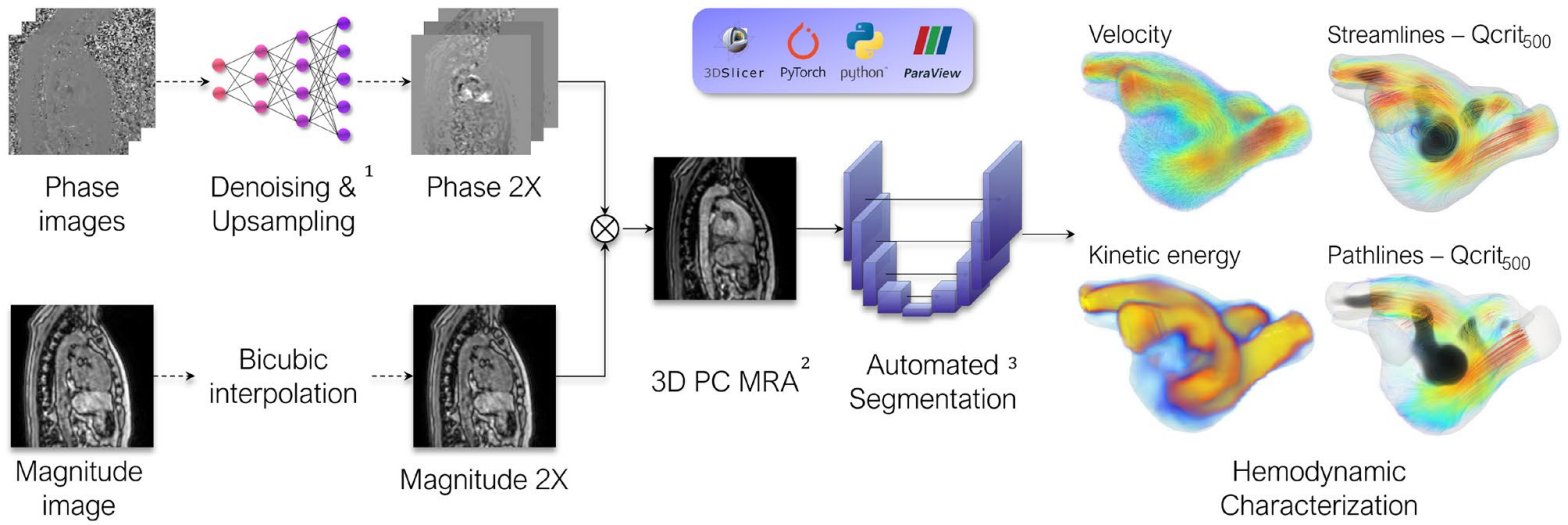
Overview of the computational framework for the advanced analysis of 4D flow magnetic resonance imaging of the left atrium. Only data from lower magnetic field strength acquisitions undergoes denoising and upsampling^20^. Next, a PC-MRA is computed^21^, followed by automatic segmentation^22^. The resulting segmentation mask facilitates the isolation of the structure of interest for subsequent quantitative and qualitative hemodynamic characterization. The entire pipeline relies exclusively on open-source software and code, ensuring accessibility and reproducibility. PC-MRA: Phase-contrast Magnetic Resonance Angiogram; Q-$\hbox {crit}_{500}$: Ratio of Q-criterion > 500 $\hbox {s}^{-2}$. Images created using ParaView (version 5.13.3) and 3D Slicer (version 5.9.0).

The developed computational pipeline (see Fig. 1) was applied to a multicenter cohort of 68 patients with varying degrees of LVDD, including healthy controls, hypertensive patients, and patients with hypertrophic cardiomyopathy (HCM) to explore the potential prognostic value of advanced 4D Flow MRI hemodynamic parameters.

## Results

### Population characteristics

**Table 1:** Study population of the quantitative analysis: Controls; HCM: Hypertrophic cardiomyopathy with no left ventricular diastolic dysfunction (LVDD); G1: HCM with grade I LVDD; G2: HCM with grade II LVDD; G2 - SAM: HCM with grade II LVDD and systolic anterior motion of the mitral valve; Hypertensive.

	Controls	HCM	G1	G2	G2 - SAM	Hypertensive
N = 68	19	9	5	6	4	25
Age (years)	36.3 ± 2.6	42.9 ± 5.1	58.4 ± 7.2	52.1 ± 7.01	66.8 ± 3.7	54.4 ± 1.1
Sex (M%)	65	70	20	50	75	56
BSA(Dubois) ($m^2$)	1.93 ± 0.06	1.92 ± 0.04	2.05 ± 0.07	1.91 ± 0.13	1.78 ± 0.11	1.93 ± 0.04
Diabetes (%)	0	10	20	16.67	0	0
Hypertension (%)	0	50	80	66.67	50	84.38
SAM (M%)	0	0	0	0	100	0
LAV (ml)	60.4 ± 3.2	80.7 ± 7.5	116.9 ± 15.9	103.1 ± 24.1	150.5 ± 23.9	97.9 ± 4.6
LVEDV (ml)	86.1 ± 5.3	74.5 ± 7.7	99.1 ± 4.1	68.4 ± 4.4	49.5 ± 4.6	75.3 ± 3.6
$\hbox {LVMass}_i$ ($g/m^2$)	62.8 ± 2.4	75.8 ± 6.1	74.3 ± 6.6	76.2 ± 11.8	87.3 ± 4.1	56.0 ± 1.9
LVEF (%)	64.1 ± 0.9	63.4 ± 2.1	59.1 ± 6.7	56.9 ± 3.5	58.3 ± 5.1	60.1 ± 0.9

Although all patients were included during segmentation training, only those who underwent TTE and had a defined LVDD degree of LVDD were included in the biomarker quantitative and qualitative analysis. Controls and hypertensive patients with indeterminate or non-zero LVDD grades were excluded, yielding a final cohort of 68 patients. HCM patients were classified by LVDD grade as follows: no LVDD (HCM), grade I LVDD (G1), and grade II LVDD (G2). G2 patients were further subdivided based on the presence of systolic anterior motion (G2-SAM), as the associated mitral valve regurgitation sufficiently alters LA hemodynamics to warrant separate analysis. Patient clinical characteristics are summarized in Table 1.

### Segmentation

**Fig. 2:**
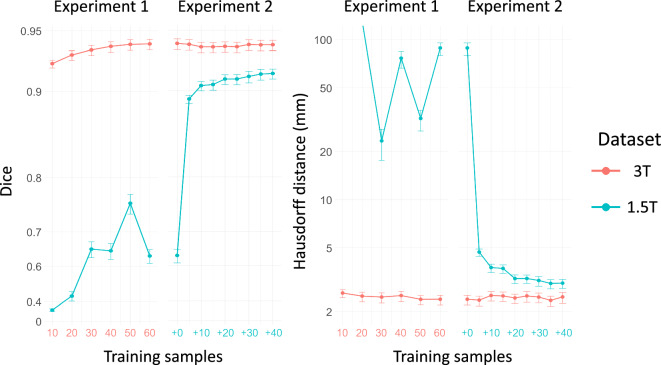
Dice score and Hausdorff 95 distance (mm) for the segmentation experiments of the left atrium. For Experiment 1, the x-axis is the total number of training cases from dataset 3 T (in red), while in Experiment 2, it is the amount of 1.5 T cases (in blue) added on top of the complete 3 T training dataset.

Figure 2 illustrates segmentation accuracy across both experiments, with representative contour visualizations provided in Supplementary Fig. S3. Experiment 1 yielded an average Dice score of 0.924 and an HD95 distance of 2.61 mm for 3 T cases from the outset, with minimal improvement from more training data. Conversely, 1.5 T cases showed poor accuracy in both metrics, with only marginal Dice score improvement despite increased training data. In Experiment 2, accuracy remained stable for the 3 T dataset. The inclusion of just five 1.5 T training cases significantly boosted the average Dice score from 0.635 to 0.892 and HD95 distance from 88.5 mm to 4.70 mm in low-resolution data. Although performance improved with additional 1.5 T training cases, it still lagged behind the accuracy in the 3 T data.

### Left atrial volume

**Fig. 3:**
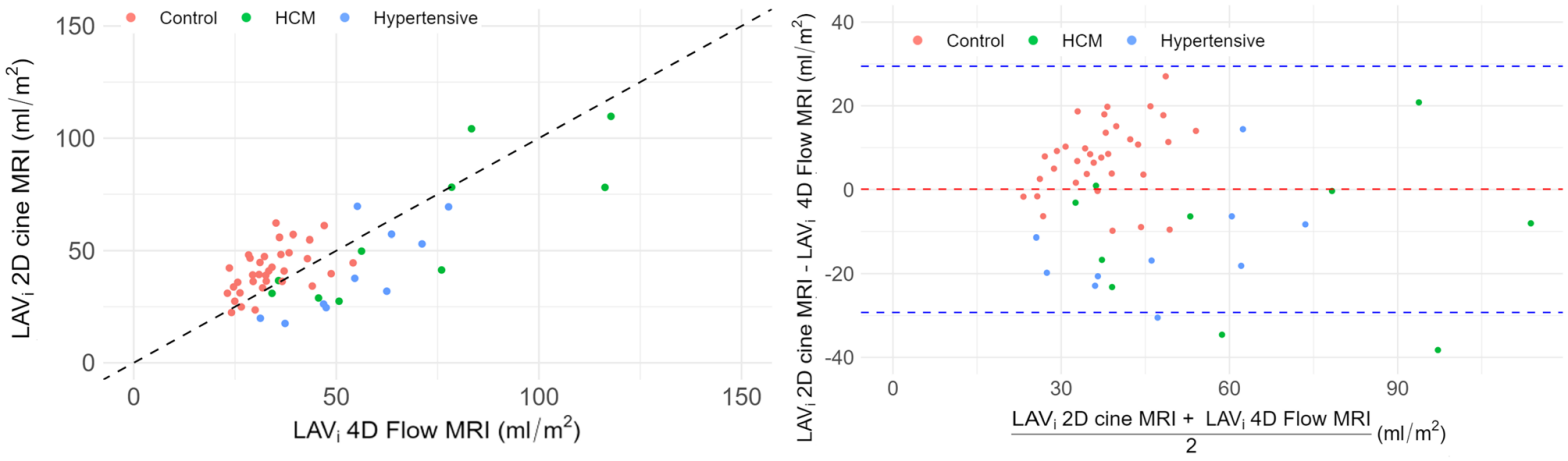
Comparison of the maximal left atrium volume indexed by body surface area ($\hbox {LAV}_i$) derived from 2D cine and 4D flow magnetic resonance imaging. On top: scatter plot with the identity line for reference. Bottom: Bland-Altman plot with the bias as a dashed red line, while the 95% confidence intervals are represented by dashed blue lines.

The $\hbox {LAV}_{i}$ results are shown in Fig. 3. A strong correlation (Pearson correlation coefficient = 0.75, 95% confidence interval 0.57-0.86) was observed between the 2D cine MRI-derived biplane method and the PC-MRA-based $\hbox {LAV}_{i}$. Bland-Altman analysis revealed a bias of 0.1 ml/$\hbox {m}^2$ and 95% limits of agreement (LoA) of (-29.3, 29.4). Cohort-specific biases emerged as evident in Fig. 3. Controls showed a positive bias of 6.7 ml/$\hbox {m}^2$ (LoA: -12.9 to 26.3 ml/$\hbox {m}^2$), and pathological cases a negative bias of -12.5 ml/$\hbox {m}^2$ (LoA: -41.8 to 16.8 ml/$\hbox {m}^2$).

### Flow rate

**Figure 4:**
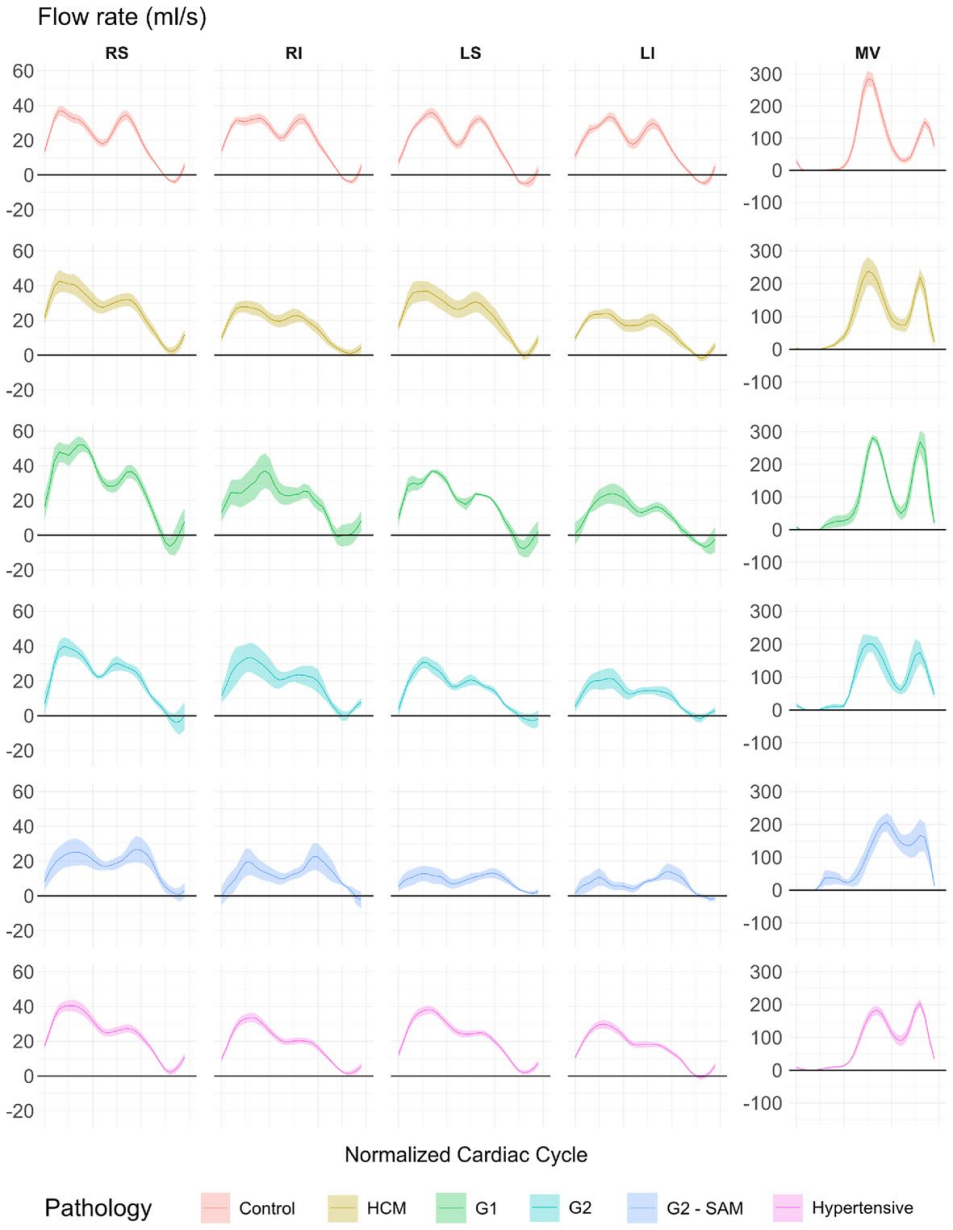
Mean and standard error of the mean of the flow rate (*ml*/*s*) of the four pulmonary veins and the mitral valve. RS: Right superior; RI: Right inferior; LS: Left superior; LI: Left inferior; MV: Mitral valve; HCM: Hypertrophic cardiomyopathy with no left ventricular diastolic dysfunction (LVDD); G1: HCM with grade I LVDD; G2: HCM with grade II LVDD; G2 - SAM: HCM with grade II LVDD and systolic anterior motion of the mitral valve.

Figure 4 displays average flow rates (ml/s) for the 4 PVs and MV across pathology groups; peak values are detailed in Table S4. The total mitral volume expelled during passive and active filling was also computed.

The relationship between the PVs varies by pathology. Healthy patients show balanced PV flow peaks, whereas pathological cases often exhibit the lowest flow in the left inferior (LI) PV. Only left PVs show significant inter-group differences during the S wave, as shown in Table S4. Although not significant post hoc, G2 SAM patients demonstrate notably reduced flow in both left PVs. a reduction that persists in the D wave. No significant differences were observed in the D wave, but left PV flow remains reduced in G2 - SAM patients.

The S/D ratio is generally stable across the four PVs, except in G2 - SAM patients. In the rest of pathological groups, the ratio is significantly higher than in healthy controls, with the highest values in G1 and hypertensive patients. ANCOVA is significant for all PVs except the right superior. For peak PV reversal flow (Ar), all groups except G1 show significantly lower peak values than controls across all PVs, independent of age. MV E/A ratios differ significantly whether based on peak flow or total volume and are not linearly related.

### Energy quantification

**Fig. 5:**
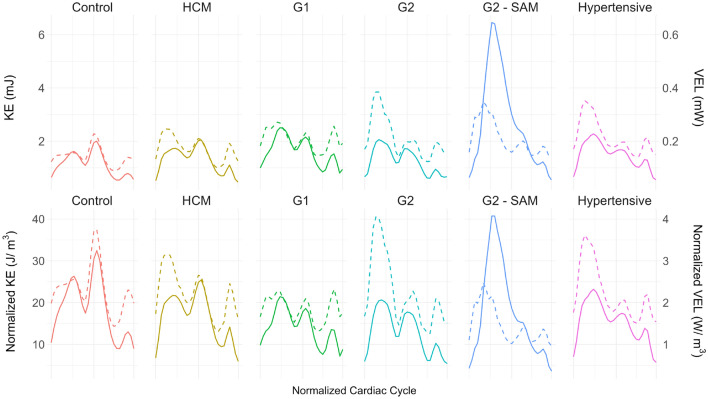
Top: Mean kinetic energy (KE) as the solid line and mean viscous energy loss (VEL) as the dashed line in the left atrium (LA). Bottom: KE and VEL normalized by LA volume. HCM: Hypertrophic cardiomyopathy with no left ventricular diastolic dysfunction (LVDD); G1: HCM with grade I LVDD; G2: HCM with grade II LVDD; G2 - SAM: HCM with grade II LVDD and systolic anterior motion of the mitral valve.

Mean kinetic energy (KE) and viscous energy loss (VEL) values are shown in Fig. 5. When LA volume, average KE per unit volume equalizes across groups, though its cardiac cycle distribution varies. Both parameters show triphasic behavior, with an initial systolic peak related to pulmonary inflow during the S wave, followed by passive and active filling.

In healthy subjects and HCM patients, early diastolic KE predominates, despite reduced systolic and E wave peaks in the latter. Other pathological groups show a shift towards a predominant systolic KE peak due to decreased early diastolic KE. Table S1, indicates significantly higher systolic peak values in G2 patients with SAM. While E wave KE differences exist across groups, these are age-dependent according to ANCOVA. Lastly, the A wave KE peak is lower in all Grade II LVDD groups.

Regarding VEL, the early diastolic peak predominates in controls. However, the systolic peak already predominates in HCM, contrary to KE. HCM, G2, and hypertensive cohorts exhibit elevated systolic VEL. Both systolic VEL and KE/VEL ratios show the most significant differences with a considerable effect size. Controls, G1, and especially G2-SAM patients display the highest values.

**Fig. 6:**
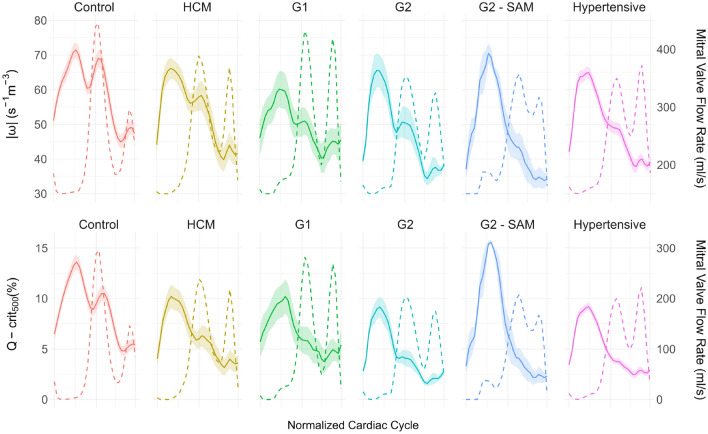
Top: Mean vorticity magnitude (solid line) and standard error of the mean with mitral valve (MV) flow rate (dashed line) for temporal reference. Bottom: Ratio of voxels in the left atrium with Q-criterion > 500 $\hbox {s}^{-2}$ (solid line), indicating vortex core areas. HCM: Hypertrophic cardiomyopathy without left ventricular diastolic dysfunction (LVDD); G1: HCM with grade I LVDD; G2: HCM with grade II LVDD; G2 - SAM: HCM with grade II LVDD and systolic anterior motion of the MV.

### Vorticity

Figure 6 shows the temporal evolution of vorticity and the Q-criterion. Both measurements exhibit three peaks, though the A peak is almost negligible. All metrics, except systolic $|\omega _{LA}|$, are statistically significant, age-independent, and have substantial effect sizes (Table S2). G2 - SAM and control groups display the highest systolic vorticity, particularly in Q-$\hbox {crit}_{500}$. In controls, early systolic and diastolic $|\omega _{LA}|$ peaks are identical, whereas Q-$\hbox {crit}_{500}$ shows a noticeable gap. This gap is present in $|\omega _{LA}|$ for other cohorts but remains more prominent in Q-$\hbox {crit}_{500}$ The early diastolic peak is delayed relative to the E wave, peaking as the passive filling decelerates and it weakens progressively with LVDD severity. Vorticity is significantly lower during atrial contraction, with only G1 patients showing values comparable to controls.

### Relative pressure $\Delta P$

**Fig. 7:**
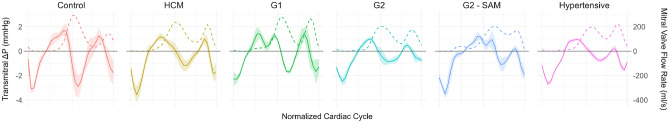
Mean and standard error of the mean of the relative pressure (mmHg) between the left atrium and left ventricle, measured using the vWERP method (solid line), accompanied by the mitral valve flow rate (dashed line). HCM: Hypertrophic cardiomyopathy with no left ventricular diastolic dysfunction (LVDD); G1: HCM with grade I LVDD; G2: HCM with grade II LVDD; G2 - SAM: HCM with grade II LVDD and systolic anterior motion of the mitral valve.

The atrioventricular pressure gradient is shown alongside the mitral flow in Figure 7. Positive gradient values indicate higher LA pressure, while negative values signify higher LV pressure. Diastole shows two positive and two negative peaks, corresponding to the acceleration and deceleration of the E and A wave, respectively. Notably, the saddle points align with the zero crossings of the relative pressure curve.

Both E peak acceleration ($\Delta \hbox {E}_{max}$) and deceleration ($\Delta \hbox {E}_{min}$), exhibit age-independent statistically significant differences with a large effect size (Table S3). Controls exhibit the highest pressure gradients. $\Delta$E*max*lacks a consistent pattern across pathological groups, though G1 and G2-SAM patients tend to have slightly higher values. Some LVDD groups, show a jagged $\Delta \hbox {E}_{max}$, without a well-defined single maxima. The start of the E wave acceleration is also delayed in G2 - SAM patients. $\Delta \hbox {E}_{min}$ is also the highest in controls and progressively decreases with worsening LVDD. The A wave acceleration peak ($\Delta \hbox {A}_{max}$) mirrors vorticity, with higher values in controls and G1 patients. No significant differences are observed in the A wave deceleration peak ($\Delta \hbox {A}_{min}$).

### Visualization

**Fig. 8:**
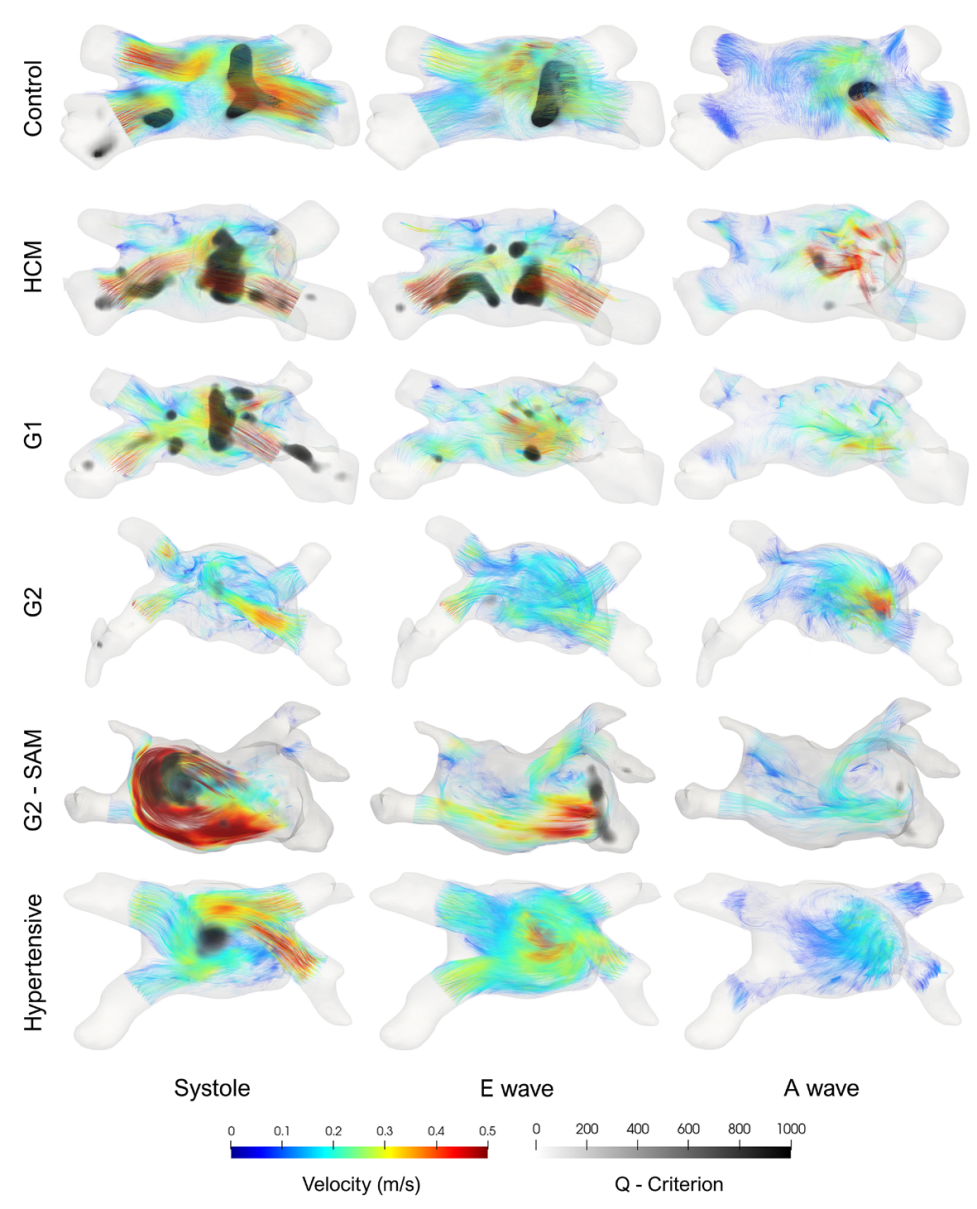
An axial view of the central left atrial vortex across diverse cardiac disorders. The visualization combines the pathlines of particles emitted from the pulmonary veins, color-coded by velocity, and a volumetric rendering of the Q-criterion, indicating the core of the vortices. HCM: Hypertrophic cardiomyopathy with no left ventricular diastolic dysfunction (LVDD); G1: HCM with grade I LVDD; G2: HCM with grade II LVDD; G2 - SAM: HCM with grade II LVDD and systolic anterior motion of the mitral valve. Images created using ParaView (version 5.13.3).

Figure 8 depicts the central LA vortex for one representative of each cohort at three points during the cardiac cycle aligning with Q-$\hbox {crit}_{500}$ peaks described above. The evolution of the vortex closely resembles the progression of the group averages shown in Fig. 6. The vortex is strongest during systole across all patients but is notably weakened in G2 patients and particularly strong in G2 patients with SAM. After the E wave, the vortex remains robust in the control and is still noticeable in the HCM patient but disappears in the remaining pathological cases. No coherent vortical structures are observed during atrial contraction.

## Discussion

This study introduces a novel 4D Flow MRI analysis framework focused on the LA. We demonstrated the feasibility of automatic neural network segmentation of the LA over 4D Flow MRI-derived PC-MRA, enabling a comprehensive quantitative and qualitative analysis of novel 4D flow MRI indices in the LA across a diverse, multicenter patient cohort, marking the first time their values were reported across a large cohort of patients with varying degrees of LVDD.

This pipeline aims to lay the groundwork for standardizing 4D Flow MRI analysis in the LA, allowing faster and more reproducible studies. Our framework was designed to balance flexibility with usability, moving beyond commercial software to allow the implementation of novel LA flow metrics while still providing tools to minimize the learning curve. Although users still need minimum proficiency in programming and 3D software, by standardizing data handling, offering template functions and dockerization, and automating most repetitive steps, we aimed to reduce technical barriers and ensure reproducibility across sites. With neural network–based segmentation, a full case can now be analyzed in under 10 minutes, making large-scale studies increasingly feasible.

The results shown in Fig. 2 evidence that a small dataset with high-quality annotations may suffice to automate 4D Flow MRI segmentation. This aligns with prior studies indicating neural networks achieve accurate segmentation with limited data^23^, challenging the presumed advantages of alternative approaches such as atlas-based segmentation^24^.

Experiment 2 underscores the adaptability of the network across diverse datasets. Despite originating from a different vendor, center, and protocol, and undergoing upsampling-denoising, adding only five 1.5 T cases to the training dataset significantly improved segmentation accuracy without compromising the performance on the 3 T data. In the context of 4D Flow MRI, where data is often limited, this approach could substantially expedite the compilation of multicenter datasets for more comprehensive clinical studies.

Turning to volumetric assessment, a strong correlation with minimal bias was observed between 2D cine and 4D Flow MRI-derived $\hbox {LAV}_i$, aligning with previous comparisons between the biplane method and 3D imaging modalities^25^. However, the bias differed significantly between control and pathological groups. This discrepancy may stem from the fact that 2D cine images are not always aligned with the LA axes, whereas 4D Flow MRI, due to its 3D nature, is not affected by such misalignment. Moreover, the geometric assumptions of the biplane method do not account for remodeling-induced changes in LA shape^11^. Similar underestimation of LA volumes in pathological cases using the biplane method has been reported in patients with atrial fibrillation^25^.

Although not its primary purpose, we have shown that 4D Flow MRI can reasonably estimate LA volume. However, further studies are needed to validate its accuracy against reference 3D sequences like CT. While our focus was on $\hbox {LAV}_{i}$ given the use of a static PC-MRA, time-resolved segmentation could enable analysis of LA function by tracking volume changes throughout the cardiac cycle.Despite its limitations in spatiotemporal resolution, 4D Flow MRI remains unique in its ability to simultaneously evaluate LA size, function, and hemodynamics. Although the limitations of 2D methods are widely known, the demand for specialized expertise and software has hindered wider clinical adoption of 3D modalities. We postulate frameworks like ours can foster broader acceptance.

In terms of hemodynamic quantification, unlike conventional TTE, 4D Flow MRI can quantify flow rates and volumes without assuming a specific flow profile, making it more robust and less sensitive to noise. Notably, E/A ratios derived from flow rates have been shown to outperform TTE in evaluating LVDD^26^. Furthermore, a key limitation of using peak velocities is that they do not account for changes in vessel cross-sectional area. For instance, PV distensibility can be affected by pulmonary circulation alterations or postural changes^27^, which impact the maximum observed velocity, whereas the flow rate remains constant.

The statistical analysis in Table S4 shows significant differences in peak E/A ratios between flow rates and total volumes. While flow rates are robust, they are single-snapshot measurements. Volumes, however, capture the entire filling phase, providing a comprehensive LVDD assessment by reflecting the passive-to-active filling ratio.

We hypothesize that PV flow rates may also offer more reliable measurements for parameters like S and D peaks, S/D ratio, and pulmonary reversal flows compared to conventional peak velocity measurements. The absence of S/D ratios less than one likely indicates a limited number of advanced LVDD cases in the dataset. Notably, the RS vein shows the least variation between across pathologies, while the other three PVs, seldom measured with TTE, exhibit the most statistically significant differences and largest effect sizes. This raises the question of whether clinically relevant diagnostic information is being overlooked by focusing exclusively on the RS PV in routine practice^11^. Finally, patients with SAM exhibit a marked difference between the left and right PVs during the S and D wave, supporting the hypothesis that the postolaterally directed mitral regurgitation jet obstructs left pulmonary inflow.

With respect to energy quantification, Fig. 5 shows results consistent with previously reported KE temporal evolution in the LA^15,28,29^ in a limited number of healthy subjects and HCM patients. In healthy subjects, and to a lesser extent in HCM patients without LVDD, the early diastolic peak reflects a compliant LV. In the remaining four groups, the KE curve shifts toward a predominant systolic peak. This shift is most pronounced in SAM patients, where mitral regurgitation induces a characteristic rotational flow pattern (Fig. 8). In Grade I and II LVDD and hypertensive patients, the systolic KE predominance appears related to an increased S/D ratio.

The KE/VEL ratio reflects flow efficiency, with higher values indicating greater energy use for transport and less loss to friction. The systolic increase in this ratio in both controls and G2-SAM patients may relate to elevated vorticity, as rotational flow better preserves KE^28^. However, the higher systolic efficiency in G1 compared to HCM or G2 patients remains unclear. No significant group differences were seen during diastole, but the marked decline from E wave to A wave suggests that active filling is less efficient at conserving momentum, beyond the energy required for atrial contraction.

Regarding vorticity, knowledge of rotational flow in the LA dates back to 2001^1^. Vortices are thought to preserve momentum^28^ and may help prevent thrombus formation^16^. Importantly, vortex structures are sensitive markers of heart disease, making the study of vorticity highly compelling for detecting early changes in LA function^16,30,31^.

The optimal approach to detect vortical structures remains contentious in fluid dynamics^32^, including in 4D Flow MRI. A key consideration is whether to normalize vorticity metrics by total volume, as simply summing vorticity across all voxels biases results in patients with dilated LAs. For instance,^16^ reported a positive correlation between vortex size and both age and LA remodeling, even though LA function typically declines with age. Similarly, Spartera et al.^18^ found that AF patients had larger vortex sizes compared to those in sinus rhythm, despite the weakened S wave and absence of diastasis in fibrillation patients, the only phases in the cardiac cycle where the atrial vortex is visible. In addition, although often not explicitly stated^33,34^, it is also common to measure vorticity based on magnitude alone, which can be misleading. Increased velocity along a non-straight flow path will invariably elevate the magnitude of vorticity, introducing a spurious term unrelated to the presence of a vortex.

Parameters such as the Q-criterion and $\lambda _2$ are explicitly designed to address these limitations and distinguish rotational from strain-dominated regions. More specifically, the Q-criterion identifies vorticity-dominant areas and enables the visualization of different scale structures by adjusting the threshold value, where a higher threshold emphasizes large-scale vortex cores. We empirically chose a threshold of 500 based on Fig. 8, with consistent results for all values above 100.

Discrepancies observed between Q-$\hbox {Crit}_{500}$ and $|\omega _{LA}|$, particularly during early filling, likely stem from the aforementioned velocity-driven term. Supporting this, we found that peak $|\omega _{LA}|$ occurs, on average, 15 ms earlier and is closer in time to the E wave peak. Visualizations in Fig. 8 also align more closely with Q-$\hbox {Crit}_{500}$ curves in Fig. 6. For instance, although G2 patients with SAM exhibit the most visually intense rotational flow during systole as a result of mitral regurgitation, their $abs{omega_{LA}}$ is not higher than that of controls. Likewise, $|\omega _{LA}|$ fails to capture the progressive weakening of vorticity from systole to early diastole as effectively as Q-$\hbox {Crit}_{500}$.

Vortex formation in the LA is primarily attributed to PV inflow collisions. Our findings reinforce this notion, with vorticity peaks aligning with both pulmonary flow maxima. Conversely, vorticity drops significantly during atrial contraction, which actively opposes pulmonary inflow.

By utilizing a specialized vortex metric normalized by the LA volume, we effectively quantified LA vorticity dynamics in a manner consistent with previous qualitative studies^30^. Most importantly, vorticity measurements, and particularly the Q-$\hbox {Crit}_{500}$, exhibit statistically significant differences between groups, irrespective of age. The early diastolic vorticity peak, in particular, shows potential as a non-invasive marker of LVDD, as it appears to gradually deteriorate with worsening diastolic dysfunction.

Lastly, while transmitral relative pressure had been previously estimated using vWERP in 4D Flow MRI data^35^, the study was limited to a small cohort with a single LVDD patient^35^. Our study provides the first comprehensive evaluation of vWERP in the left heart across a larger, more diverse LVDD patient population. As expected, healthy subjects show the highest $\Delta \hbox {E}_{max}$ and $\Delta \hbox {E}_{min}$, indicating a compliant LV with efficient relaxation. Elevated $\Delta \hbox {E}_{max}$ in G2-SAM patients may be linked to mitral regurgitation, which distends the LA and delays passive filling, resulting in a steeper initial pressure gradient.

In our cohort, $\Delta \hbox {E}_{min}$ appears to better reflect LVDD progression than $\Delta \hbox {E}_{max}$. $\Delta \hbox {E}_{min}$ can be considered analogous to the TTE-derived mitral flow deceleration time (DT)^36^. Unlike DT, which is not recommended in patients with E and A wave fusion (e.g., hypertensives and G2-SAM patients in our study), $\Delta \hbox {E}_{min}$ can readily identify the end of the E wave where the pressure gradient crosses zero, as shown in Fig. 7.

$\Delta \hbox {A}_{max}$ shows the expected progression across LVDD stages in HCM patients. An initial significant increase from HCM to G1 suggests compensatory atrial contractility for impaired relaxation. Afterward, $\Delta \hbox {A}_{max}$ progressively decreases with deteriorating atrial function in G2 and G2-SAM patients.

Although vWERP and hemodynamic forces^37^ offer promising alternatives to invasive catheterization, they remain proxies for absolute pressures. Nonlinear E and A wave changes during LVDD progression can create ambiguity, especially without knowledge of absolute LA or LV filling pressures. This complicates differentiation between LVDD grades or between healthy individuals and those with pseudonormal filling. Thus, the clinical impact and predictive power of 4D Flow MRI-derived atrioventricular pressure gradients require further dedicated evaluation.

A major limitation of this study is the lack of temporally resolved segmentation. While dynamic manual segmentation using time-resolved PC-MRA from^21^ is likely feasible during high-velocity cardiac intervals, the low signal-to-noise ratio in the LA during the remaining intervals makes single-step segmentation challenging and irreproducible. Moreover, the annotation workload would would also increase by 20-30 fold. Consequently, previous attempts have relied on manually segmented end-diastolic and end-systolic frames, combined with non-rigid registration to estimate remaining frames, raising concerns about intermediate timestep accuracy^24^. Incorporating 4D convolutions to make the segmentation networks aware of temporal consistency, along with the use of sparse loss functions, could reduce the manual segmentation burden to just 20% of the total dataset^38^, while improving intermediate timestep prediction.

Registration of 4D Flow MRI to sequences with better myocardium-to-blood contrast sequences, such as 3D bSSFP MRI, is another viable alternative^39^. However, given that bSSFP is not always available and considering the overarching goal of integrating multicenter data, we chose to focus on the magnitude and phase images, which are an intrinsic part of 4D Flow MRI acquisitions. Finally, time-resolved segmentation would also require additional preprocessing, such as the need to track the mitral valve and the four PVs for accurate flow rate measurement^40^.

Another limitation is related to acquisition orientation. In our study, all images were obtained in axial orientation, and oblique or sagittal acquisitions were not specifically evaluated. Although the nnU-Net preprocessing step automatically resamples all images to a common voxel spacing and reorients them into a standardized reference frame, thereby reducing the impact of acquisition orientation^22^, the network’s performance on non-axial orientations remains to be formally validated.

Time-averaged segmentation can include static tissue, violating Navier-Stokes continuity assumptions and increasing vWERP estimation error. However, vWERP has proven reliable for assessing intracardiac pressure gradients in dynamic flow domains^35^, provided the static mask approximates an intersection segmentation (i.e., remains within the flow domain throughout). Time-averaged PC-MRA is biased toward regions of signal accumulation, i.e., constant flow, where vWERP remains accurate. Minor segmentation dilations have negligible impact on pressure gradient estimation^35,41^, even when some static tissue is included at the edges.

The datasets from both centers included in this study were not were not specifically tailored for the low-velocity atrial environment, but rather for broader cardiovascular assessment, meaning that the VENC of the acquisition was higher than ideal for atrial flow quantification. Nevertheless, a strength of our framework is that it still yielded high-quality segmentations and statistically significant differences between patient groups even when data are not optimally tailored to the LA. Future studies may benefit from acquisition protocols specifically tailored for atrial hemodynamics, such as dual-VENC or adaptive VENC approaches, which can help improving velocity-to-noise ratio (VNR) without compromising the analysis of other cardiac chambers and major vessels^42^. In addition, the 1.5 T data were acquired during free breathing without explicit respiratory motion correction. Navigator-gating, retrospective image registration, and quality assurance were applied to mitigate motion effects; however, residual respiratory motion may still affect intra-atrial velocities, especially near the pulmonary veins.

Regarding 4D Flow MRI data denoising and upsampling, 4DFlowNet was trained with synthetic CFD data from the aorta and tested on single-center, single-magnetic-field-strength in vivo data. Ideally, 4DFlowNet should be validated with paired low- and high-resolution 4D Flow MRI acquisitions in the LA, but such data are challenging to obtain. Retraining with domain-specific CFD simulations can reduce prediction error^43^. However, despite advances in wall motion boundary conditions based on dynamic CT^44^, LA CFD simulations are not yet realistic enough to offer significant advantages over the original network^45^.

Caution is warranted when interpreting the statistical analysis due to small cohort sizes, particularly across LVDD grades. The primary aim of this preliminary study is to demonstrate the framework’s potential, report benchmark values for novel 4D Flow MRI indices in the LA, and lay the foundation for future research. Publicly releasing the entire process aims to facilitate larger 4D flow MRI dataset collection in the LA, which could corroborate or challenge our findings. Notably, the most statistically significant differences were observed in parameters exclusive to 4D flow MRI. Existing non-invasive guidelines for the assessment of LVDD have a sensitivity as low as 35%^46^, underscoring the need to identify new parameters for assessing diastolic relaxation abnormalities independently of confounding factors such as age^4^.

However, given the heterogeneity in LVDD etiology, it is unlikely that a single parameter will provide a definitive diagnosis. Moreover, much of the quantitative analysis in this study, and most clinical guidelines, rely on measuring discrete peak values from inherently time-resolved signals at specific cardiac cycle points. Moreover, modalities like 4D flow MRI generate vast amounts of data that exceed human interpretation capabilities. Unsupervised machine learning methods could better integrate such data, as they excel at identifying underlying patterns in time-resolved, high-dimensional datasets to generate interpretable and clinically relevant phenogroups^47^.

## Conclusions

In the present study, we present a novel computational pipeline for the comprehensive analysis of multiple center 4D Flow MRI data in the LA. Segmentation was fully automated, leveraging well-established neural networks, and generalizing to data from two distinct centers, even with a limited number of training cases. Furthermore, a comprehensive analysis of novel 4D Flow MRI indices in the LA was conducted in a diverse cohort with varying LVDD degrees. The results demonstrate the potential of 4D Flow MRI to provide a more comprehensive assessment of LA hemodynamics compared to conventional echocardiographic methods, particularly in the context of LVDD. By making the entire framework open-source, we hope to encourage further research into 4D Flow MRI-based characterization of LA hemodynamics by contributing to the standardization and acceleration of its analysis.

## Methods

Figure 1 provides a high-level overview of the framework. First, lower-resolution acquisitions undergo denoising and upsampling. Afterward, the phase and magnitude images are combined to generate a time-averaged phase-contrast magnetic resonance angiogram (PC-MRA) that is automatically segmented. The resulting segmentation is then used to mask the flow data, which is transformed into a format suitable for subsequent qualitative and quantitative analysis.

### Population and Data Acquisition

To showcase the versatility of the pipeline, we analyzed a dataset comprising 109 subjects, incorporating data from two centers. The Centre of Advanced MRI (CAMRI) at the University of Auckland, New Zealand, recruited 39 healthy participants. Additionally, we included 29 patients with HCM and varying degrees of LVDD, and 41 hypertensive patients from the Department of Cardiology, Hospital Clínic de Barcelona, Spain. By including healthy controls together with patients spanning HCM, hypertension, and a range of diastolic dysfunction, we aimed to capture a representative spectrum of left atrial remodeling and hemodynamic perturbation. This allowed us to assess whether advanced flow metrics derived from 4D flow MRI differ across common clinical phenotypes, thereby providing a foundation for evaluating their potential prognostic value in future studies. Ethical approval for the healthy volunteer cohort was obtained from the Health and Disability Ethics Committee New Zealand (17/CEN/226), and written informed consent was obtained from all participants. Ethical approval for the patient cohort was granted by the Clinical Research Ethics Committee of the Hospital Clínic de Barcelona (HCB/2015/0455 and HCB/2022/0207), and written informed consent was obtained from all participants. All methods were performed in accordance with the Declaration of Helsinki and all relevant guidelines and regulations.

The CAMRI data were acquired using a Siemens Magnetom 1.5 T Avanto Fit (Siemens Healthcare, Erlangen, Germany) scanner with the following acquisition parameters: axial slab orientation, retrospective cardiac gating, free breathing, 30-channel coil array, velocity encoding = 150 cm/s, flip angle = $7^{\circ }$, echo time = 2.3 ms, repetition time = 38.8 ms, voxel size = 2.4 x 2.375 x 2.375 mm acquired and reconstructed, a field of view of 125 mm x 285 mm x 380 $\hbox {mm}^{3}$, acquisition matrix 52 x 110 x 160, 20 phases reconstructed, parallel imaging with an acceleration factor of 3 with an average scan time of 8 minutes and no contrast used. The remaining data were obtained with a SIGNA Architect 3.0T MRI scanner (General Electric Medical Systems) featuring an axial slab orientation, retrospective cardiac gating, respiration compensation 10% of k-space, small anterior 16-channel and spine posterior 40-channel coil array, velocity encoding = 160 cm/s, a flip angle of $15^{\circ }$, an echo time of 2.216 ms, a repetition time of 4.172 ms, a voxel size of 2.2 mm x 2.2 mm x 2.2 mm acquired and 1.4063 mm x 1.40635 mm x 1.1 mm reconstructed, a field of view of 360 x 360 x 176 $\hbox {mm}^{3}$, acquisition matrix 160 x 160 x 90, 30 phases reconstructed, HyperKat acceleration with a factor of 8, an average scan time of 10 minutes and the use of contrast (Gadoterate meglumine 0.1 mmol/kg). Hereafter, we will denote the datasets from the Siemens and General Electric scanners as datasets 1.5 T and 3 T, respectively. Processing of the raw phase data, including background phase offset correction and velocity anti-aliasing, was done at the acquisition centers with Arterys, which applies automatic phase unwrapping using a piecewise linear polynomial model with Gaussian smoothing, combined with AI-based semi-automatic static tissue detection^48^.

In addition, 2D balanced steady-state free precession (bSSFP) cine MRI images of the two-chamber and four-chamber views were acquired for a total of 42 patients, including 21 patients from the 1.5 T cohort and 21 from the 3 T cohort. Furthermore, 88 patients (23 controls, 27 with HCM, and 38 with hypertension) underwent PW Doppler velocimetry via TTE in the apical four-chamber view and were assessed for echocardiographic LVDD grading based on the 2016 ASE/EACVI guidelines^36^.

### Data denoising and upsampling

Diverse magnetic field strengths, acquisition protocols, and vendors result in 4D Flow MRI data of differing spatial resolution and quality across centers. This can have a substantial impact on the geometry and quantitative measurements of velocity^49^. As it is often necessary to pool data from several centers to gather a sufficiently large dataset for clinical studies, it is imperative to minimize the differences in spatial resolution and image quality to reduce measurement bias.

In this regard, we applied the 4DFlowNet super-resolution network^20^ to simultaneously denoise and upsample the phase images by a factor of two. As the neural network was only trained to upsample the phase image, the resolution of the magnitude image was doubled through bicubic interpolation. Only data from the 1.5 T scanner was upsampled using the available pre-trained model, resulting in a final spatial resolution of 1.2 mm x 1.1875 mm x 1.1875 mm, closely matching the 3 T dataset.

### Segmentation

Precise segmentation of the region of interest significantly enhances visual qualitative analysis and enables the quantification of clinically relevant parameters. Nevertheless, segmentation remains a challenging aspect in 4D Flow MRI analysis, particularly in the LA, primarily due to its low spatial resolution^50^. The time-consuming nature of manual segmentation further hinders the broader adoption of 4D Flow MRI in clinical settings^51^.

#### PC-MRA

4D Flow MRI segmentation commonly involves generating a phase-contrast magnetic resonance angiogram (PC-MRA). In the resulting image, phase data yields higher intensities in areas of high blood flow, while the magnitude image adds morphological information and mitigates noise in regions with low signal, such as the lungs. We utilized the equation proposed by^21^, which incorporates a correction factor $\gamma < 1$ to enhance low-velocity areas. While the original article considered a time-resolved PC-MRA, segmentation of the LA in timesteps of low velocities proved infeasible, as shown in Supplementary Fig. S2. Consequently, we adopted a time-averaged version of the equation introduced by^21^:

1 $$\begin{aligned} \text {PC-MRA} = \frac{1}{N} \sum _{t=1}^{N} M(t) \cdot ({V_x}^{2}(t)+{V_y}^{2}(t)+{V_z}^{2}(t))^{\gamma }\,, \end{aligned}$$

where *M*(*t*) is the signal magnitude and ${V_x}$, ${V_y}$, and ${V_z}$ are the three components of the velocity. After qualitative visual testing, we selected $\gamma =0.4$ for the LA. The resulting PC-MRA from the different values of $\gamma$ are provided in the Supplementary Figure S1 alongside examples of both good and poor-quality single-timestep PC-MRA.

**Fig. 9:**
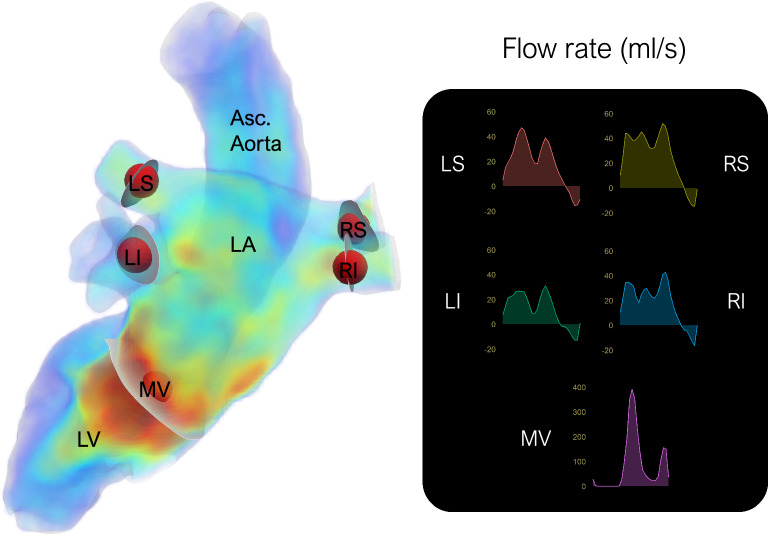
A volumetric rendering of the 4D flow magnetic resonance imaging velocity field in the left heart is shown, encompassing the left atrium, left ventricle, and ascending aorta. During processing, spheres are manually placed within regions of interest to serve as sample volumes to determine the dominant flow direction and define vessel cross-sections (shown in grey) for flow rate computations. On the right, the corresponding flow rates (*ml*/*s*) are shown for the mitral valve and the four pulmonary veins. LA: Left atrium, LV: Left ventricle, Asc. Aorta: Ascending aorta, MV: Mitral valve, RS: Right superior, LS: Left superior, RI: Right inferior, LI: Left inferior. Images created using ParaView (version 5.13.3).

#### Automatic segmentation of the left atrium

Besides being time-consuming, manual segmentation entails a high degree of dependence on the annotator’s experience and is prone to intra- and inter-observer variability. Consequently, the segmentation was automated using the nnU-Net^22^, which has already proven effective in segmenting aortic PC-MRA^51^. The nnU-Net is a self-adaptive framework that automates the selection of optimal preprocessing, data augmentation, network architecture, and postprocessing strategies. This ensures maximum accuracy with minimal user input for each segmentation task. Training was completed on an Nvidia RTX A6000 GPU. The training dataset was manually segmented in Slicer3D by an experienced annotator, with the PVs segmented until the first bifurcation. To test the capabilities of the network on 4D flow MRI data, two separate experiments were conducted.

First, we tried to determine the minimum number of cases needed to effectively train the network on a single dataset. For this purpose, we separated 60 cases from the 3 T scanner and performed successive runs, adding 10 more cases to the training dataset in each iteration. In the second experiment, we sought to find the minimum number of ground truth cases required to generalize to a new dataset. We used the 60 cases from the 3 T scanner as the starting point and successively added 5 cases from the 1.5 T dataset to the training dataset in each run. Ten cases from each dataset were allocated for testing. The performance of each model was reported in terms of the Dice score and the 95th percentile of the Hausdorff distance (HD95). The nnU-Net was trained for 1000 epochs, taking around 70 hours to complete a single fold on average. Despite the primary focus of the pipeline being on the LA, segmentation of the LV and ascending aorta was also incorporated in separate networks for visualization and computing the pressure gradients.

### Quantitative and qualitative analysis

To extract meaningful knowledge from the high-dimensional 4D Flow MRI data, it is crucial to generate a comprehensive set of descriptive and easily understandable quantitative and qualitative metrics. In the following section, we include a detailed description of all the quantitative indices and visualizations included in our framework. We then perform a comprehensive comparison of these parameters in a diverse cohort of patients to establish their potential prognostic significance in the LA, serving as the groundwork for future studies.

#### Segmentation post-processing

After segmentation, the region of interest is isolated by zeroing all velocities outside the segmentation mask. The data is then converted to VTK image format (.vti) for use in ParaView. The segmentation mask is also converted to a triangular surface mesh (.stl) and a tetrahedral volumetric mesh (.vtk) for volume and flow rate computations. The whole process is semi-automated using Python code and ParaView state files. User input is limited to the manual placement of the spheres on the structures to be measured, as shown in Fig. 9, which includes the four PVs and the MV. These spheres serve as the origin for the vessel cross-sections. The normal vector of the cross-section plane is determined by averaging the flow direction during the five timesteps with the highest velocity magnitude across the cardiac cycle.

#### Left atrial volume

The indexed LA volume ($\hbox {LAV}_{i}$) is a robust prognostic marker of poor cardiovascular outcomes^52^ and an indicator of the severity and chronicity of LVDD^53^. Segmentation of the 4D Flow MRI-derived PC-MRA provides a volume estimate without the geometric assumptions of 2D imaging approaches^54^. To evaluate the accuracy of 4D Flow MRI derived LAV*i* measurements, we conducted a direct comparison with the biplane disk summation method from 2D cine MRI^55^.

#### Hemodynamic indices

Although conventional flow parameters (e.g., peak velocity, LA volume) remain the most widely used in routine practice, emerging evidence suggests that advanced hemodynamic descriptors could provide unique insights into LA pathophysiology not reflected by traditional indices^5^. While research into their utility in the LA is gaining traction^5,56^, substantial gaps remain in our understanding. To help address these uncertainties, our framework incorporates as many of these parameters as possible, as listed in the following:

**Flow rate:** This is the volume of blood passing through a given area per unit of time. In our framework, the flow rate is measured directly from the vessel cross-sections generated in ParaView. Unlike velocity, it provides information about the movement of blood volume between the different chambers without being influenced by variations in vessel cross-section.

**Kinetic energy:** KE in the LA quantifies the intracavitary energy content of blood motion and thus reflects the efficiency of atrioventricular coupling^5,29^. Reduced LA KE has been observed in paroxysmal AF and heart failure with preserved ejection fraction even in the absence of apparent remodeling or strain abnormalities, and has been shown to predict AF recurrence after cardioversion^17,57,58^. KE may therefore serve as an integrative marker of atrial function and energy transfer, with potential prognostic implications. The KE was calculated as:

2 $$\begin{aligned} KE = \frac{1}{2} m v^2 = \frac{\rho V_{tot}}{2}\sum _{i=1}^{N} v_{i}^2 \,, \end{aligned}$$

where *m* is the mass, computed as the product of the density $\rho$ and the total volume $V_{tot}$ of the region of interest, multiplied by the summation of the velocity magnitude $v_{i}$ in each voxel.

**Viscous energy loss:** This is the energy lost due to the frictional forces (or viscosity) within the blood flow. It can help in understanding the efficiency of blood flow within the LA^5^. The viscous dissipation, $\phi _v$, can be computed using the Navier-Stokes energy equations^59^:

3 $$\begin{aligned} \Phi _v= & \frac{1}{2} \sum _{i} \sum _{j} \left[ \left( \frac{\partial v_i}{\partial x_j} + \frac{\partial v_j}{\partial x_i} \right) - \frac{2}{3} \left( \nabla \cdot \textbf{v} \right) \delta _{ij} \right] \end{aligned}$$

4 $$\begin{aligned} \delta _{ij}= & {\left\{ \begin{array}{ll} 1 & \text {for } i = j \\ 0 & \text {for } i \ne j , \end{array}\right. } \end{aligned}$$

where the first term (Equation 3) represents the symmetric part of the velocity gradient tensor and the second the divergence of the velocity field. Finally, the temporal variation of the viscous energy loss ($EL_t$) can be computed as follows:

5 $$\begin{aligned} EL_t = \mu \sum _{i=1}^{N} \Phi _v \text {Vol}_i \,, \end{aligned}$$

where $\mu =$0.0035 Pa $\cdot$ s is the viscosity of the blood, $\hbox {Vol}_i$ is the voxel volume, and *N* is the number of voxels.

**Vorticity:** Vorticity is a measure of the rotation of the velocity field. Vortical structures naturally form in the LA and LV during filling, where they play an important role in preserving momentum and optimizing blood transport with minimal energy loss^1^. In the LV, vorticity has been shown to be a sensitive and reproducible marker of LVDD^18,33^. In the LA, however, vortex formation can be disrupted by atrial remodeling, which promotes flow stasis and impairs washout, and has been associated with an increased risk of thrombus formation and embolic brain infarcts in AF patients^34^. The curl, or vorticity, is defined by:

6 $$\begin{aligned} \boldsymbol{\omega } = \nabla \times \textbf{v}\,, \end{aligned}$$

where $\nabla$ is the curl operator and $\textbf{v}$ is the velocity field. Since the equation produces a vector field, it is commonplace to compute its magnitude, $|\omega |$, for better interpretability. Additionally, we normalized $|\omega |$ by the LA volume ($|\omega _{LA}|$) to mitigate the bias from a dilated LA. However, $|\omega |$ solely serves as a measure of the fluid’s rotation rate and may be problematic in regions dominated by shear flow^32^. For this reason, we have incorporated the Q-criterion^60^, a parameter explicitly engineered for the detection of vortex cores:

7 $$\begin{aligned} Q = \frac{1}{2} \left( \left| \left| \boldsymbol{\Omega }\right| \right| ^2 - \left| \left| \boldsymbol{S}\right| \right| ^2\right) , \end{aligned}$$

where $\boldsymbol{\Omega }$ denotes the vorticity tensor and $\textbf{S}$ is the strain-rate tensor. Unlike vorticity, the Q-criterion yields a scalar field, where positive values indicate regions dominated by rotational motion, while negative values mark areas where viscous forces dominate.

Various thresholds can be selected for the Q-criterion to visualize vortical structures at varying scales. A threshold of Q-criterion > 500 $\hbox {s}^{-2}$ was quantitatively determined based on visualizations in Fig.  8. To normalize by the LA volume, we calculated the ratio of all voxels within the LA with Q-criterion > 500 $\hbox {s}^{-2}$ (Q-$\hbox {crit}_{500}$).

**Relative pressure** $\Delta P$: Pressure gradients describe the underlying functional driver of blood flow, and as such, the evaluation of local pressure gradient abnormalities is an integral part of several guidelines for gauging cardiac function. LA pressure is the cornerstone determinant of pulmonary venous hypertension and LV filling pressures, with elevated LA pressure being a hallmark of diastolic dysfunction that correlates directly with symptoms and clinical outcomes^36^. Although still in an exploratory phase, atrioventricular pressure gradients have been postulated as a potential marker for left heart function^35,61^.

Since 4D Flow MRI provides a complete description of the velocity field, pressure gradients can be derived without the constraints and assumptions required in other non-invasive modalities^35^. Amidst the plethora of available methods, we opted for the virtual work-energy relative pressure (vWERP), which utilizes a work-energy formulation of the Navier-Stokes equation to extract pressure gradients through arbitrary vascular^62^ and cardiac structures^35^.

#### Statistical analysis

To complement the visualizations of the temporal evolution of each hemodynamic metric, a comprehensive statistical analysis of the most relevant maxima and minima was conducted to better assess their significance. In line with the longstanding goal of identifying age-independent biomarkers for non-invasive LVDD assessment, an analysis of covariance (ANCOVA) was performed to determine significant differences between cohorts while controlling for age. Mean values, along with the statistical significance (P-value) and strength of the association (effect size, $\eta ^2$) for each cohort, are provided in the Supplementary Information. If intergroup differences were found to be statistically significant ($\alpha <0.05$), a post hoc analysis was conducted using a pairwise Tukey test and Cohen’s D to quantify the effect size. A Benjamini-Hochberg correction for multiple comparisons was also applied in the post hoc tests.

#### Visualization

The interpretation of intricate flow patterns and phenomena within the heart chambers can pose a considerable challenge. To leverage the wealth of information provided by 4D Flow MRI data, it is imperative to develop appropriate visualizations. Once the case is segmented and the spheres have been placed in the structures of interest, our pipeline automatically generates several interactive visualizations in ParaView, including volumetric renderings, vector maps, streamlines, and pathlines.

As an example of its potential clinical utility, we used one of the automatically generated visualizations to study the evolution of vortical structures in the LA, as illustrated in Fig. 8. This 3D representation combines a pathline visualization of the velocity and a volumetric rendering of the Q-criterion. The pathline visualization provides a detailed portrayal of flow patterns and velocities within the LA. Particles are emitted from the PV at each time step, tracing the path of each particle for the preceding 6 time steps, color-coded by velocity. The volumetric renderings of the Q-criterion approximate the location of the vortex cores. A median filter with an isotropic 3-voxel kernel was applied to eliminate noise and accentuate the largest vortex cores.

Lastly, all the visualizations generated in our pipeline can be easily exported as pre-rendered animations through ParaView. It can then be easily uploaded as a .zip file to web-based visualization platforms such as ParaView Glance which enables easy interaction with the 3D scene without requiring any previous experience in handling visualization software.

## Supplementary Information

Supplementary Information.

## Data Availability

The code for the computational pipeline, along with the weights for the segmentation neural network, are publicly available at the following repository: https://github.com/Xtaltec/LA-4D-Flow-MRI.
