# Supplementary material for: Automated 4D flow MRI pipeline for the quantification of advanced hemodynamic parameters in the left atrium
Source: Sci Rep. 2026 Jan 16;16:5426. doi: 10.1038/s41598-025-34972-7 (PMC12886947; doi:10.1038/s41598-025-34972-7)
Supplement: Supplementary file 1 — Supplementary Information. [file 41598_2025_34972_MOESM1_ESM.pdf]

Supplementary Information

S1 Statistical analysis results

Table S1. Average values and standard error of the mean of the kinetic energy (KE) and viscous energy loss (VEL) peaks, and the ratio between them (KE/VEL) for the different pathologies. The ANCOVA P-values and effect size (η_p^2) results are shown for the effect of pathology and age as a covariable. The P-values are color-coded, ranging from green (0.0) to red (0.2), indicating the level of statistical significance. Similarly, η_p^2 is represented using a color gradient, ranging from white (0.0) to blue (0.4) for pathology and white (0.0) to red (0.4) for age, indicating the strength of the association. Lastly, the posthoc results are shown for the pairwise comparisons between pathologies if they are found to be statistically significant ($\alpha < 0.5$).

		Pathology						ANCOVA: P-value		ANCOVA: η_p^2		Posthoc
		Control	HCM	G1	G2	G2 - SAM	Hypertensive	Pathology	Age	Pathology	Age	Tukey and Cohen's D
KE_{LA} (J/m^3)	S	29.11 ± 2.61	24.39 ± 2.08	21.85 ± 3.89	22.1 ± 2.39	42.26 ± 4.92	25.57 ± 1.29	.005	.34	.233	.015	G1 <G2 - SAM; P = .045; D = -2.22, G2 <G2 - SAM; P = .045; D = -2.65, G2 - SAM >HCM; P = .045; D = 2.42, G2 - SAM >Hypertensive; P = .045; D = 2.36
	E	40.9 ± 3.28	29.92 ± 5.48	19.16 ± 1.57	21.43 ± 3.16	16.39 ± 3.74	23.31 ± 1.5	.197	.0	.109	.277	
	A	18.91 ± 1.63	16.81 ± 3.42	16.41 ± 2.69	13.23 ± 1.65	9.44 ± 2.02	14.63 ± 1.36	.006	.003	.228	.134	
VEL_{LA} (W/m^3)	S	2.85 ± 0.22	3.43 ± 0.32	2.35 ± 0.37	4.48 ± 0.7	2.6 ± 0.35	4.18 ± 0.38	.016	.569	.197	.005	
	E	4.71 ± 0.32	3.31 ± 0.41	2.15 ± 0.31	2.62 ± 0.37	1.54 ± 0.35	2.78 ± 0.23	.029	.002	.178	.139	G1 <N; P = .005; D = -1.93, G2 <N; P = .022; D = -1.57, G2 - SAM <N; P = .001; D = -2.35, Hypertensive <N; P = .0; D = -1.48
	A	3.06 ± 0.25	2.54 ± 0.5	2.64 ± 0.51	2.49 ± 0.46	1.5 ± 0.35	2.47 ± 0.18	.002	.001	.252	.161	
KE/VEL	S	10.47 ± 0.8	7.3 ± 0.45	9.82 ± 1.63	5.4 ± 0.71	18.43 ± 5.57	7.14 ± 0.56	.0	.715	.392	.002	G1 <G2 - SAM; P = .041; D = -1.1, G2 <G2 - SAM; P = .0; D = -1.87, G2 - SAM >HCM; P = .0; D = 1.88, G2 - SAM >Hypertensive; P = .0; D = 2.49, G2 - SAM >N; P = .013; D = 1.51
	E	8.88 ± 0.67	8.82 ± 0.78	9.97 ± 2.12	8.38 ± 0.83	10.76 ± 1.57	8.99 ± 0.47	.407	.089	.077	.046	
	A	6.29 ± 0.34	6.76 ± 1.0	6.5 ± 0.89	5.78 ± 0.71	6.68 ± 1.0	6.07 ± 0.32	.92	.728	.022	.002	

Table S2. Average peak values and standard error of the mean for the vorticity magnitude normalized by the left atrium volume ($|\omega_{LA}|$) and the ratio of voxels with Q-Criterion above 500 s^{-2} (Q-Crit$_{500}$). The ANCOVA P-values and effect size (η_p^2) results are shown for the effect of pathology and age as a covariable. The P-values are color-coded, ranging from green (0.0) to red (0.2), indicating the level of statistical significance. Similarly, η_p^2 is represented using a color gradient, ranging from white (0.0) to blue (0.4) for Pathology and white (0.0) to red (0.4) for age, indicating the strength of the association. Lastly, the posthoc results are shown for the pairwise comparisons between pathologies if they are found to be statistically significant ($\alpha < 0.5$).

		Pathology						ANCOVA: P-value		ANCOVA: η_p^2		Posthoc
		Control	HCM	G1	G2	G2 - SAM	Hypertensive	Pathology	Age	Pathology	Age	Tukey and Cohen's D
$\|\omega\|$ $(s^{-1}m^{-3})$	S	73.35 ± 2.52	68.59 ± 3.16	61.42 ± 5.0	67.27 ± 4.76	71.98 ± 3.24	68.84 ± 1.85	.546	.082	.061	.048	
	E	73.84 ± 2.48	60.89 ± 4.31	51.94 ± 3.86	53.13 ± 4.38	45.24 ± 5.06	53.29 ± 1.75	.007	.121	.221	.092	G1 <N; P = .004; D = -2.04, G2 <N; P = .004; D = -1.88, G2 SAM <N; P = .0; D = -2.61, Hypertensive <N; P = .0; D = -2.06
	A	53.3 ± 1.99	45.7 ± 3.67	48.79 ± 4.6	42.29 ± 1.27	36.44 ± 2.4	42.16 ± 1.39	.0	.094	.312	.044	G2 - SAM <N; P = .037; D = -1.99, Hypertensive <N; P = .004; D = -1.39
Q-crit$_{500}$ (%)	S	14.2 ± 0.8	11.1 ± 1.2	10.5 ± 1.6	9.6 ± 1.0	15.9 ± 0.4	9.8 ± 0.4	.0	.108	.339	.041	G2 - SAM >Hypertensive; P = .018; D = 2.83, Hypertensive <N; P = .0; D = -1.59
	E	11.9 ± 0.8	6.9 ± 1.1	6.2 ± 1.2	4.3 ± 0.8	6.7 ± 2.2	4.9 ± 0.4	.0	.167	.369	.085	G1 <N; P = .014; D = -1.59, G2 <N; P = .0; D = -2.23, HCM <N; P = .007; D = -1.36, Hypertensive <N; P = .0; D = -2.45
	A	6.3 ± 0.5	4.4 ± 0.9	5.7 ± 1.5	3.3 ± 0.3	2.9 ± 0.6	3.4 ± 0.3	.001	.186	.279	.028	Hypertensive <N; P = .006; D = -1.41

Table S3. Average peak values and standard error of the mean for the vWERP-derived relative pressure (mmHg) measurements. The ANCOVA P-values and effect size (η_p^2) results are shown for the effect of pathology and age as a covariable. The P-values are color-coded, ranging from green (0.0) to red (0.2), indicating the level of statistical significance. Similarly, η_p^2 is represented using a color gradient, ranging from white (0.0) to blue (0.4) for Pathology and white (0.0) to red (0.4) for age, indicating the strength of the association. Lastly, the posthoc results are shown for the pairwise comparisons between pathologies if they are found to be statistically significant ($\alpha < 0.5$).

		Pathology						ANCOVA				Posthoc
		Control	HCM	G1	G2	G2 - SAM	Hypertensive	P-value Pathology	P-value Age	η_p^2 Pathology	η_p^2 Age	Tukey and Cohen's D
ΔP (mmHg)	ΔE_{max}	3.2 ± 0.23	1.81 ± 0.33	1.99 ± 0.4	1.35 ± 0.11	1.97 ± 0.41	1.63 ± 0.09	.0	.823	.447	.001	G2 <N; P = .0; D = -2.21, HCM <N; P = .007; D = -1.52, Hypertensive <N; P = .0; D = -2.26
	ΔE_{min}	-4.53 ± 0.78	-2.03 ± 0.51	-1.81 ± 0.12	-1.51 ± 0.16	-1.2 ± 0.47	-1.8 ± 0.21	.01	.93	.234	.0	Hypertensive >N; P = .006; D = 1.24
	ΔA_{max}	2.6 ± 0.87	1.2 ± 0.44	2.26 ± 0.63	0.85 ± 0.13	0.23 ± 0.26	0.88 ± 0.12	.004	.007	.261	.123	
	ΔA_{min}	-2.66 ± 0.94	-2.33 ± 0.81	-2.52 ± 0.77	-1.2 ± 0.12	-1.99 ± 0.66	-2.15 ± 0.23	.306	.019	.104	.099	

Table S4. Average peak values and standard error of the mean for the flow rate parameters. The ANCOVA P-values and effect size (η_p^2) results are shown for the effect of pathology and age as a covariable. The P-values are color-coded, ranging from green (0.0) to red (0.2), indicating the level of statistical significance. Similarly, η_p^2 is represented using a color gradient, ranging from white (0.0) to blue (0.4) for pathology and white (0.0) to red (0.4) for age, indicating the strength of the association. Lastly, the posthoc results are shown for the pairwise comparisons between pathologies if they are found to be statistically significant ($\alpha < 0.5$).

		Pathology						ANCOVA: P-value		ANCOVA: η_p^2		Posthoc
		Control	HCM	G1	G2	G2 - SAM	Hypertensive	Pathology	Age	Pathology	Age	Tukey and Cohen's D
E (ml/s)	MV	350.71 ± 23.44	293.46 ± 42.31	290.24 ± 8.89	265.66 ± 28.6	224.81 ± 31.02	251.46 ± 16.56	.644	.003	.052	.135	
A (ml/s)	MV	191.29 ± 15.76	239.13 ± 26.78	285.92 ± 39.89	211.36 ± 34.64	184.53 ± 50.25	266.4 ± 11.41	.037	.009	.17	.105	
E_{VOL} (ml)	MV	56.63 ± 4.39	51.72 ± 8.67	57.2 ± 9.83	49.06 ± 2.55	36.7 ± 9.19	39.56 ± 3.19	.279	.001	.094	.177	
A_{VOL} (ml)	MV	18.18 ± 1.51	27.57 ± 3.34	32.58 ± 4.01	26.71 ± 3.73	28.84 ± 3.74	30.97 ± 1.64	.038	.007	.169	.112	Hypertensive >N; P = .0; D = 1.63
E/A	MV	2.15 ± 0.25	1.32 ± 0.22	1.08 ± 0.12	1.52 ± 0.35	2.3 ± 1.36	0.99 ± 0.08	.014	.015	.202	.091	Hypertensive <N; P = .018; D = -1.49
E_{VOL}/A_{VOL}	MV	3.63 ± 0.41	2.13 ± 0.44	1.89 ± 0.43	2.2 ± 0.53	1.43 ± 0.53	1.4 ± 0.12	.037	.0	.17	.279	Hypertensive <N; P = .0; D = -1.74
S (ml/s)	RS	39.53 ± 3.67	45.26 ± 6.21	57.22 ± 4.87	42.15 ± 4.58	29.0 ± 7.6	48.24 ± 3.91	.212	.981	.106	.0	
S (ml/s)	RI	38.15 ± 3.28	31.26 ± 2.8	38.07 ± 10.19	36.23 ± 9.78	21.61 ± 7.39	40.5 ± 2.9	.268	.888	.098	.0	
S (ml/s)	LS	38.82 ± 3.44	39.36 ± 6.17	39.13 ± 1.66	32.86 ± 4.01	14.47 ± 4.57	42.79 ± 2.6	.019	.908	.192	.0	
S (ml/s)	LI	36.93 ± 3.46	26.93 ± 2.94	25.14 ± 5.4	23.56 ± 5.54	11.38 ± 4.96	33.87 ± 2.62	.027	.006	.181	.115	
D (ml/s)	RS	38.88 ± 3.49	35.74 ± 3.54	37.46 ± 4.01	33.3 ± 3.51	27.57 ± 7.45	33.69 ± 2.79	.956	.039	.017	.067	
D (ml/s)	RI	37.52 ± 3.62	25.94 ± 3.76	25.07 ± 3.15	27.07 ± 4.96	22.7 ± 7.76	25.53 ± 2.2	.634	.025	.053	.079	
D (ml/s)	LS	37.57 ± 2.73	32.1 ± 6.09	24.23 ± 0.96	22.58 ± 2.88	13.23 ± 2.72	28.11 ± 1.9	.24	.029	.101	.075	
D (ml/s)	LI	34.89 ± 3.85	21.83 ± 3.75	16.83 ± 2.62	16.2 ± 3.08	14.08 ± 4.5	22.35 ± 2.23	.282	.001	.094	.158	
S/D	RS	1.09 ± 0.08	1.3 ± 0.15	1.57 ± 0.15	1.27 ± 0.05	1.13 ± 0.31	1.49 ± 0.07	.116	.013	.13	.096	
S/D	RI	1.11 ± 0.09	1.36 ± 0.17	1.43 ± 0.38	1.22 ± 0.18	0.98 ± 0.24	1.62 ± 0.08	.016	.003	.2	.138	
S/D	LS	1.08 ± 0.08	1.35 ± 0.11	1.62 ± 0.09	1.47 ± 0.09	1.14 ± 0.45	1.58 ± 0.07	.026	.044	.182	.064	Hypertensive >N; P = .01; D = 1.36
S/D	LI	1.15 ± 0.07	1.34 ± 0.1	1.45 ± 0.12	1.53 ± 0.33	0.91 ± 0.36	1.61 ± 0.09	.018	.055	.194	.058	
Ar (ml/s)	RS	-5.78 ± 1.02	1.01 ± 2.12	-10.15 ± 5.28	-7.26 ± 6.87	-1.41 ± 3.3	1.63 ± 1.75	.018	.384	.194	.012	
Ar (ml/s)	RI	-9.28 ± 1.09	-1.77 ± 1.49	-8.62 ± 4.7	0.42 ± 3.24	-3.31 ± 4.46	-0.79 ± 1.2	.002	.823	.265	.001	Hypertensive >N; P = .005; D = 1.49
Ar (ml/s)	LS	-7.25 ± 2.94	-1.43 ± 2.16	-12.04 ± 4.36	-6.08 ± 3.96	1.03 ± 1.03	-0.75 ± 1.44	.078	.574	.145	.005	
Ar (ml/s)	LI	-9.45 ± 1.19	-2.52 ± 1.7	-10.79 ± 3.53	-3.11 ± 2.18	-2.88 ± 0.87	-2.11 ± 0.99	.001	.575	.269	.005	Hypertensive >N; P = .003; D = 1.4

S2 PC-MRA: γ values and single timestep segmentation

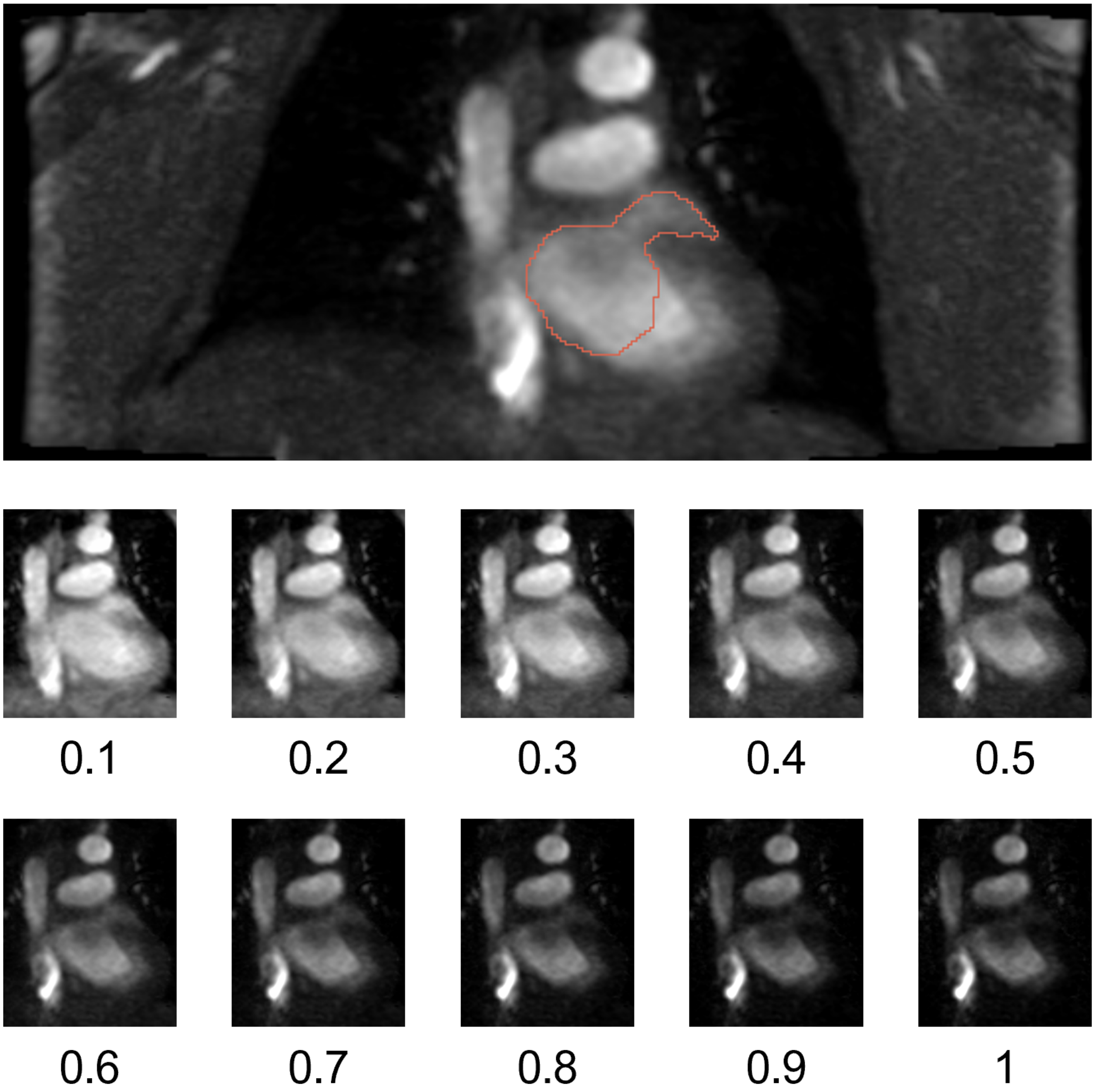

Figure S1. Three-dimensional phase-contrast magnetic resonance angiograms (PC-MRA) were generated using Equation 1. The coronal view of the chosen γ value of 0.4 is shown at the top, with the outline of the left atrium segmentation in red. Below, the resulting 3D PC-MRA for 10 different γ values are displayed. The lowest γ values produce minimal contrast between soft tissues and blood, while low-velocity structures like the left atrial appendage (LAA) become nearly indiscernible at the highest γ values. The intermediate γ values strike a balance between blood pool and tissue contrast while highlighting low-velocity regions.

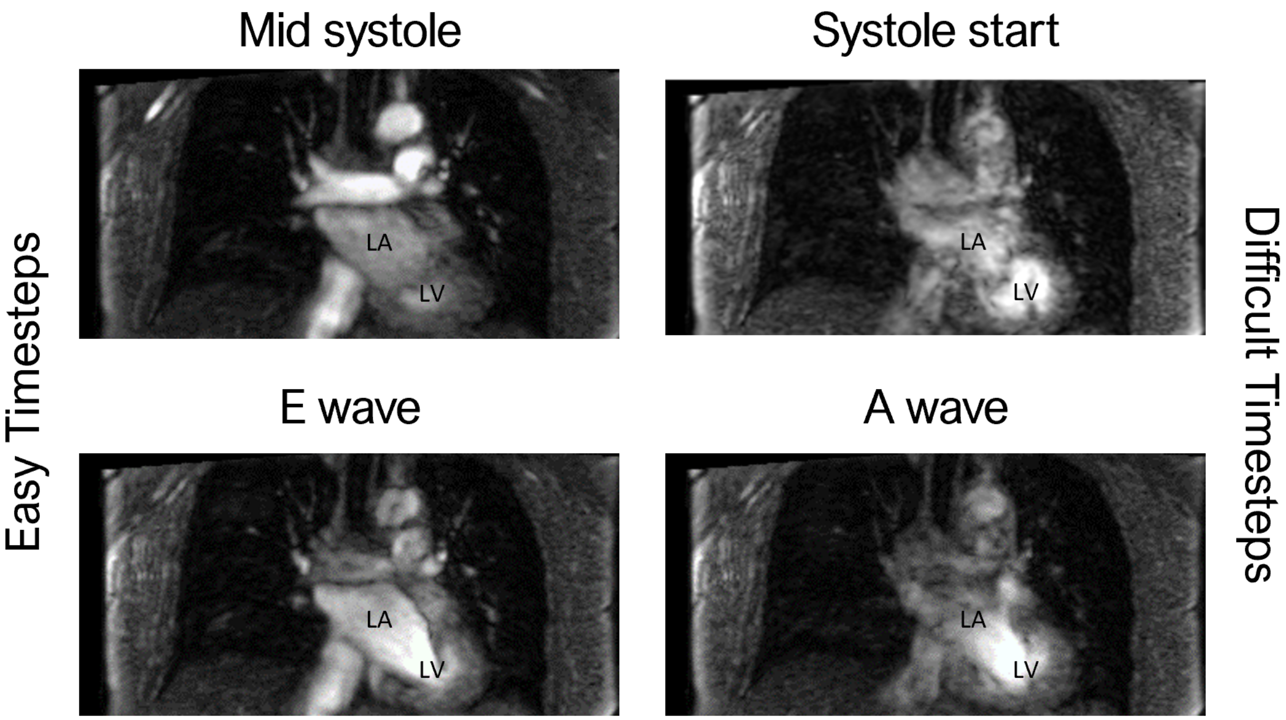

Figure S2. Coronal view of the time-resolved four-dimensional phase-contrast magnetic resonance angiograms (PC-MRA) of a hypertrophic cardiomyopathy patient, generated using the equation proposed by[21]. A $\gamma = 0.4$ was employed. During time steps when left atrial velocities are high, segmentation becomes feasible, as evidenced by the two examples in the left column, where the LA contour is clearly discernible. However, during periods of low velocity, such as the onset of the cardiac cycle or instances where high velocity is limited to specific areas (e.g., atrial contraction), distinguishing the LA from other cardiac structures might prove challenging or even infeasible.

S3 Segmentation results

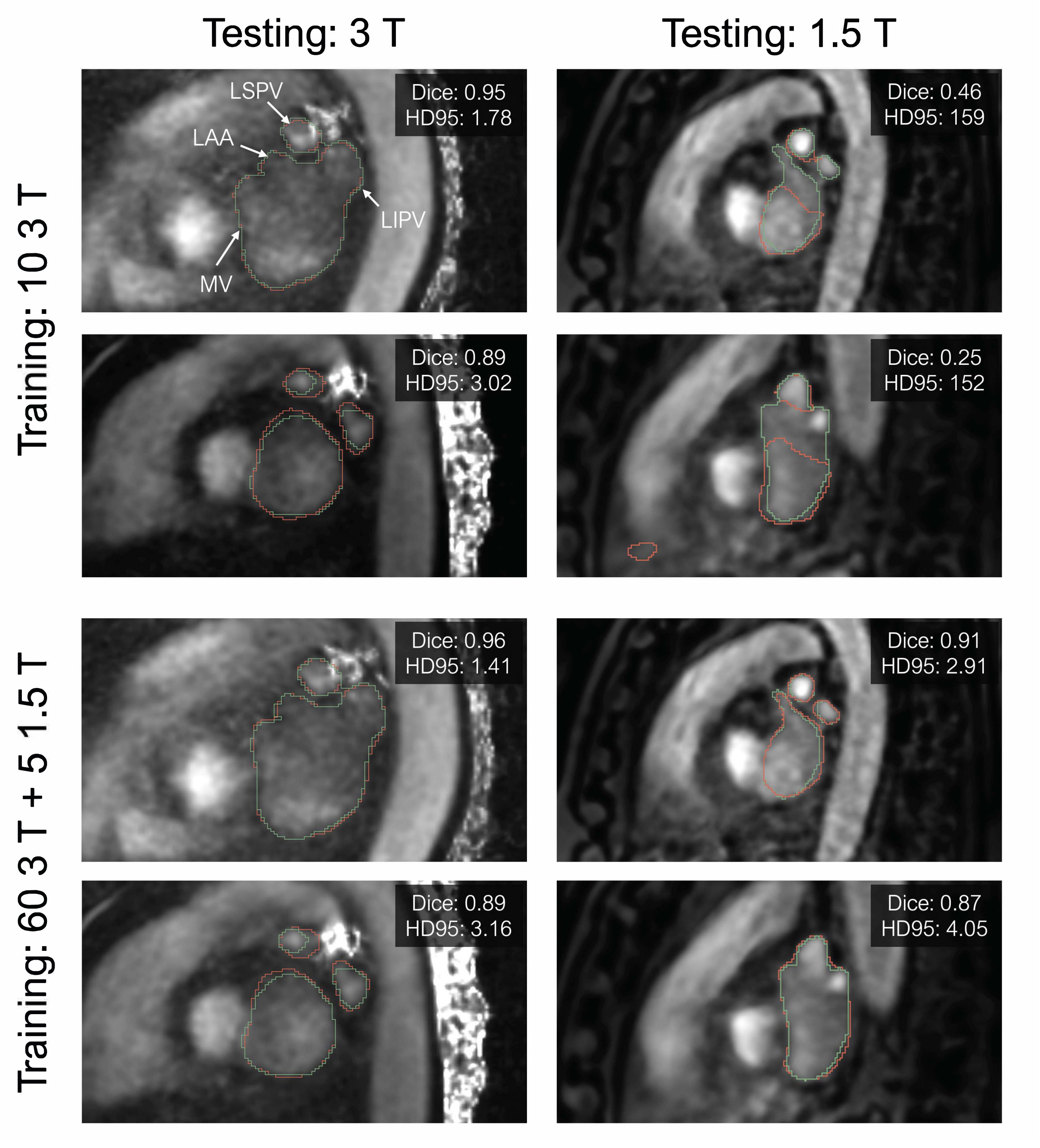

Figure S3. Contours of the manually annotated ground truth segmentations (green) and nnU-Net predictions (red) overlaid over the time-averaged phase-contrast MR angiograms (PC-MRA) in the sagittal view. The first two rows show the best and worst-performing test cases from the first iteration of Experiment 1 (10 3T during training) using 1.5T and 3T datasets. The last two rows display the same cases after training with 60 3T and 5 1.5T cases (Experiment 2), showing significant improvement in 1.5T test cases, while the 3T cases slightly improve or remain consistent. Dice: Dice score, HD95: 95th percentile Hausdorff distance (mm), MV: Mitral valve, LAA: Left atrial appendage, LSPV: Left superior pulmonary vein, LIPV: Left inferior pulmonary vein.
